# Formation and Magnetic Properties of Transition Metal Atomic Chains on Monolayer MoS_2_ Grain Boundaries: A First-Principles Study

**DOI:** 10.3390/nano14242043

**Published:** 2024-12-20

**Authors:** Zhiyuan Li, Shuqing Yang, Yiren Wang

**Affiliations:** 1 Key Laboratory for Nonferrous Materials (MOE), School of Materials Science and Engineering, Central South University, Changsha 410083, China; 8204201406@csu.edu.cn (Z.L.); 8204181110@csu.edu.cn (S.Y.); 2 National Key Laboratory for Powder Metallurgy, Central South University, Changsha 410083, China

**Keywords:** atomic chain, monolayer MoS_2_, transition metal, first principles, grain boundaries

## Abstract

Magnetic one-dimensional nanostructures show great potential in spintronics and can be used as basic building blocks for magnetic materials and devices with multiple functions. In this study, transition group atomic chains (V, Cr, Mn, Fe, Co, and Ni) are introduced into nonmagnetic MoS_2_ with a 4|8ud-type grain boundary. Based on first-principles calculations, the V atomic chains show good thermodynamic stability and can self-assemble along the grain boundary direction. The formation of V, Cr, Mn, and Ni atomic chains can induce magnetism into a 4|8ud-type MoS_2_ system through typical d-d and p-d interactions. This joint effect of transition metal doping and grain boundaries on the magnetism of monolayer MoS_2_ is of great significance for exploring the electromagnetic properties of monolayer MoS_2_ for the development of electronic devices.

## 1. Introduction

With the continuous miniaturization of device sizes, one-dimensional (1D) materials such as linear atomic chains (LACs), nanoribbons (NRs), and nanowires (NWs) have attracted considerable interest [1,2,3,4,5]. One-dimensional materials present multiple functional properties and wide applications in optics, electronics, and magnetic technologies [6,7]. Their unique low-dimensional structures and multifunctional properties make them candidates for future atomic-scale spintronic devices. The development of advanced experimental techniques such as mechanical controllable breaking junctions (MCBJs) and scanning tunneling or atomic force microscopy (STM or AFM) have prompted the realization of linear-atomic-chain-based devices through the manipulation of atoms [8]. In addition, their deposition on anisotropic substrates or step surfaces through self-assembly growth has been regarded as a powerful technique for integrating various 1D atomic chains as well [9,10]. However, introducing magnetism into the artificially created 1D structures to fabricate spintronic devices remains a technical challenge.

Many researchers have been dedicated to obtaining physically stable magnetic 1D atomic chains deposited on metallic substrates. One pioneering study on ferromagnetic monoatomic Co chains succeeded in preparing a high density of parallel atomic chains along steps. The atomic chain on a stepped Pt (111) surface was fabricated by growing Co on a high-purity Pt (997) vicinal surface through STM [11,12]. X-ray magnetic circular dichroism techniques were applied to investigate the long-range-ordered ferromagnetism of Co wires, and it was owed to the large localized orbital moments of Co and the presence of anisotropy barriers [12]. Later, Hirjibehedin [13] used STM to build magnetic chains as long as 10 Mn atoms on a thin insulating layer of CuN, and the spin excitation spectra suggested that the spin configuration and the strength can be tuned by the coupled atomic spins. These pioneering works have paved the way for the future practical design of ferromagnetic atomic chains, and theoretical studies have been performed to explore the possible systems. Hammer [14] predicted that Fe double chains on the Ir (001) surface present an antiferromagnetic state instead of a ferromagnetic state due to Fe-Ir hybridization using ab initio calculations [15]. Systematic studies of all 3D transition metal (TM) freestanding linear and zigzag chains demonstrated that they could have a stable or metastable ferromagnetic state, and a large orbital magnetic moment was found in the FM Fe, Co, and Ni linear chains [16]. In one study, it was found that the magnetic anisotropy in 3D chains became smaller as the structure moved from the linear to zigzag structure, and the magnetic moment originated from the spin split of d orbitals. Later, these researchers investigated the magnetic properties of the V, Cr, and Mn linear atomic chains on a Cu (001) surface based on density functional theory [17]. The presence of the Cu (001) substrate could stabilize the ferromagnetic state in the V linear chain on the hollow sites on Cu (001), while decreased orbital moments were calculated compared to freestanding nanowires. More recently, Zhao et al. [18] used first-principles calculations, taking black phosphorene (BP) as a prototype 2D substrate, and found that the elements Pd and Pt prefer to grow along the trough in an atom-by-atom manner. Due to the unique periodic structure of two-dimensional (2D) monolayer materials, they are considered as a potential substrate for 1D atomic chain deposition.

Monolayer MoS_2_ with graphene-like cross link structures is one of the most studied 2D materials. Large-scale monolayer MoS_2_ is generally fabricated through chemical vapor deposition (CVD) [19]. The structural defects such as point defects [20,21,22] and grain boundaries (GBs) [23,24,25] are inevitable during the CVD preparation and post-treatment process. Both intrinsic and extrinsic point defects are known to greatly influence the magnetic behaviors of the 2D MoS_2_ [26,27,28,29,30]. The GBs in MoS_2_ present a one-dimensional, regular structure with multi-atomic defect rings distributed along the GB area. Primary attempts have been made to explore the one-dimensional regular structure in MoS_2_ GBs. Electron irradiation studies of the atomic structures indicated that V_s_ gradually forms and diffuses under the bombardment of electron beam, and finally aggregates into a S vacancy chain in the single-layer MoS_2_ [31]. Energetics calculations confirmed the feasibility of MoS_2_-based catalysts since the alcohol synthesis from syngas can be more favorable for a system with a sulfur-vacancy patch [32]. Further magnetic investigations of monolayer MoS_2_ by substituting a linear S vacancy with transition metal atoms (V, Cr, Mn, and Fe) found that linearly V doping can induce magnetic moments of 3.714 μ_B_ [33]. First-principles calculations have shown that GB models with a single homo-elemental bond are ferromagnetic, with magnetic moments of 0.32–1.10 μ_B_/nm originating from the unpaired 4d electrons of the Mo atoms in the vicinity of defect rings [25]. Preliminary studies have revealed that the single Ni and Mn atoms prefer occupying the interstitial site of the octatomic ring of the GB due to the lattice misfit strain and can induce potential ferromagnetism into the MoS_2_ [34]. To investigate the feasibility of extending atomic chains and to enable the opening of additional magnetic windows within the system, the formation of atomic chains and the associated changes in magnetism based on single-atom vacancies are explored. These one-dimensional structural defects on the two-dimensional monolayer can provide a potential growing environment of one-dimensional atomic chains.

Although the magnetic properties of MoS_2_ with point defects or grain boundaries alone have been intensively reported [20,21,22,23,24,25], there are few studies on the joint effect of grain boundary doping. The GBs possess much more open structures than the pristine monolayer structure, which enables the formation of dopant atomic chains. In this study, a single-layer MoS_2_ model with a 4|8ud grain boundary was constructed and doped with various transition metal elements (including V, Cr, Mn, Fe, Co, and Ni), hereinafter referred to as the TM/GB@MoS_2_ system. First-principles energetics were performed to investigate the formation possibilities of one-dimensional transition metal atomic chains. Spin-polarized calculations were carried out to evaluate the electronic and magnetic properties of potential monatomic chains.

## 2. Computational Details

First-principles calculations were performed based on density functional theory (DFT) [35] with generalized gradient approximation (GGA) parameterized by Perdew, Burke, and Ernzerhof (PBE) [36]. The geometry optimizations and electronic structure calculations are performed with the projector augmented wave (PAW) pseudopotential [37], and the plane wave cutoff energy is set to be 450 eV, as implemented in the Vienna ab initio simulation package (VASP) code [38,39]. The maximum Hellmann–Feynman force between each atom during geometry optimization is less than 0.02 eV/Å, and energies are converged to within 1.0 × 10^−5^ eV per atom. Spin-polarized states are considered for all magnetic and electronic calculations. All the calculations were performed under the same relaxation criteria.

The 4|8 ring pairs grain boundary model, denoted as MoS_2__4|8ud, was built with 330 atoms with a misorientation angle of 60°, while the distance between the two grain boundaries was set to 18 Å, resulting in a crystal structure with lattice parameters a = 3.20 Å, in which the vacuum layer size is 20 Å. Due to the periodic boundary conditions, there are two identical grain boundary regions in the constructed GB model. And the results for atomic numbers up to n = 4 refer to finite chains, while the results for n = 5 refer to an infinite chain. The thermodynamic stabilities of grain boundaries with TM dopants can be evaluated by formation energy (E_f_) calculations using the following equation [27,40]:

E_f_ = (E_TM_ − E_perfect_ − N_i_ × μ_i_)/2

Here, E_TM_ and E_perfect_ stand for the total energy of the GB model with and without TM dopants, respectively. N_i_ is the number of atoms of type i that have been added into the mode, and μ_i_ represents the relevant chemical potentials of these atoms, which is taken as the energy per atom in its metallic state. The constant 2 represents the presence of two identical grain boundaries in the constructed TM/GB@MoS_2_ models. The detailed values of μ_i_, E_TM_, and E_perfect_ for each calculation are listed in Supporting Information Table S1, and the values of E_f_ are listed in Supporting Information Tables S2 and S3. Compared with the formation energy information in Supporting Information Table S1, transition metal atoms tend to form atomic chains at the grain boundaries.

## 3. Results and Discussion

### 3.1. Geometry and Band Structure of MoS_2_ Grain Boundaries

**Figure 1:**
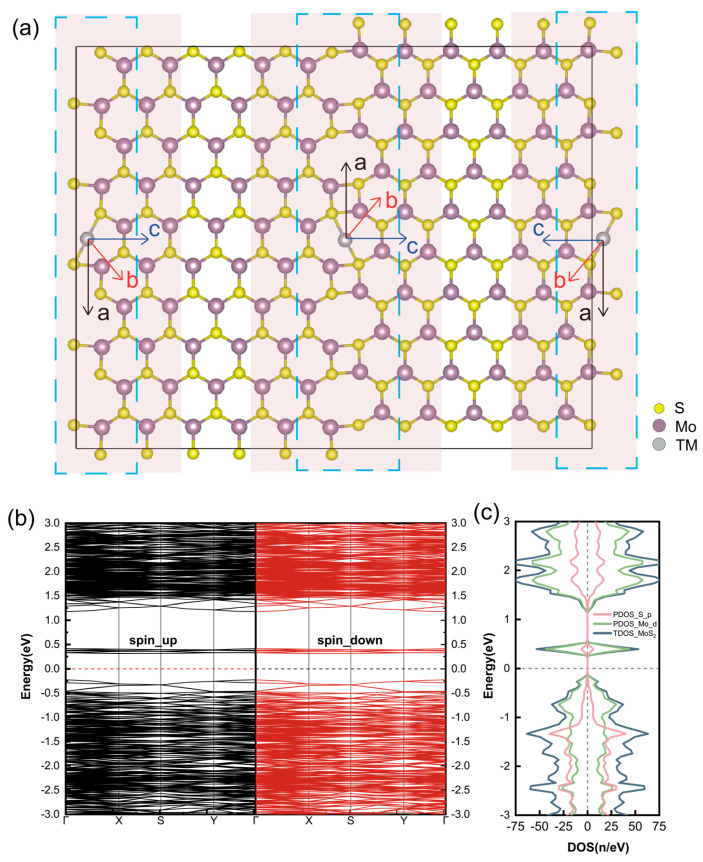
(**a**) Structural diagram of monolayer MoS_2_ grain boundary with 4|8 ring pairs and the corresponding (**b**) band structure and (**c**) density of states. The arrows in (**a**) indicate the assembly directions for the atomic chain. The atoms covered by the pink rectangle are the ones undergoing dynamic relaxation. The atoms at the grain boundary are enclosed by blue dashed lines.

The constructed grain boundary structure with 4|8 ring pairs is shown in Figure 1. The MoS_2__4|8ud GB system has been predicted to be energetically favorable with lower formation energy compared to other 60° GBs [34,41]. Monolayer MoS_2_ has a direct bandgap of ~1.70 eV, while defect bands appear with the presence of GB structures, resulting in a much narrower bandgap, which agrees well with the recent theoretical report of a 4|8 GB [42]. Experimental observations have shown decreases in the bandgap width with the formation of (5|7) and (4|6)/(6|8) GBs as well [43,44]. Moreover, the value of the bandgap can be underestimated by the adopted exchange–correlation functionals GGA-PBE [45,46]. The GB in MoS_2_ can introduce localized states within the bandgap. The symmetric distribution of the band structure and DOS in both spin directions suggests a nonmagnetic nature.

### 3.2. Geometry and Formation Energy of Adsorbed Atomic Chains on GB

The assembly of TM atomic chains on the GB starts from a single TM adsorbed on the center of the octatomic ring of the GB, as indicated by the gray ball in Figure 1. The calculated formation energies suggest that the adsorption tendency follows V > Fe > Ni > Co > Mn > Cr. Considering the fact that a single TM defect generally has a relatively high formation energy in pristine monolayer MoS_2_ [26,27], the presence of the GB can promote TM atoms to segregate from the monolayer to the GB region by thermodynamics. The defect formation energies for a single TM atom at the substitutional Mo site of the MoS_2_ GB are listed in Table S1 from reference [34]. Most of the negative formation energies refer to S-rich conditions, which implies that TM elements are favorable for substitution without the presence of the GB under an exceeded sulfur concentration. However, only single-atom substitution is considered, and the substituted sites in the GBs are limited. With increasing the TM numbers, the TM elements shall segregate from the substitutional sites to the interstitial sites of the GB once the substituted sites are saturated. Therefore, despite the fact that initial adsorption for a single TM at the GB is predicted to be endothermic, dopant segregation can initiate the assembly of the TM atomic chains on the GB.

**Figure 2:**
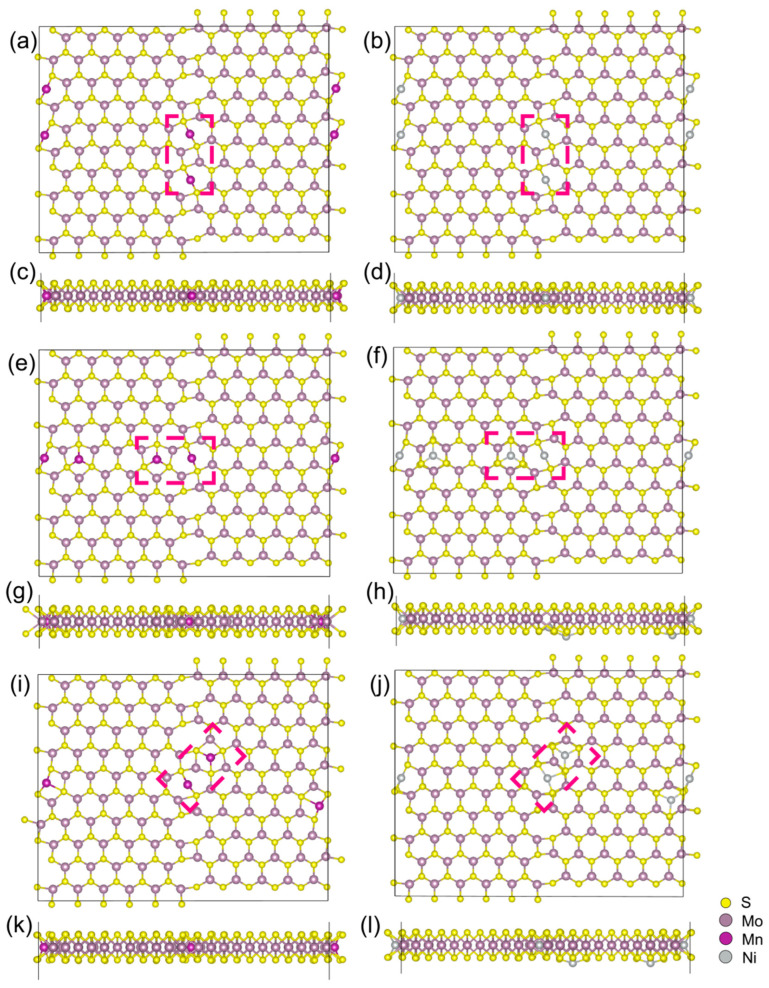
Top view and front view of the relaxed structure of TM_2_/GB@MoS_2_ self-assembled along directions a, b, and c: (**a**,**c**) are for Mn_2_/GB@MoS_2_ along the direction a; (**e**,**g**) are for Mn_2_/GB@MoS_2_ along the direction c; (**i**,**k**) are for Mn_2_/GB@MoS_2_ along the direction b; (**b**,**d**) are for Ni_2_/GB@MoS_2_ along the direction a; (**f**,**h**) are for Ni_2_/GB@MoS_2_ along the direction c; (**j**,**l**) are for Ni_2_/GB@MoS_2_ along the direction b. The transition metal atomic chains formed in different directions are enclosed by pink dashed lines.

Three possible assembly directions are considered for the second TM atom, denoted as a, b, and c in Figure 1. However, calculations for binary atomic chains along the diagonal and horizontal lines result in relatively high formation energies, as seen in Table 1. Two transition metal elements with significantly different sizes were chosen: Ni with a smaller radius (1.24 Å) and Mn with a larger radius (1.37 Å). For the case where the number of TM atoms is n = 2, the formation energies of these two TM atoms when doped along the a, b, and c directions were calculated. Large lattice distortion can be found for TM assembly along the b and c directions as well, where the TM interstitial hexatomic rings and top S atomic layers turn to repel and induce large lattice expansion, which can be obviously observed in Figure 2. The Ni atoms originally located within the hexagonal rings are pushed to the exterior of the MoS_2_ “sandwich” structure after dynamic relaxation, as shown in Figure 2h,i, beyond the surface sulfur atomic layers. The subsequent building blocks of (TM@GB)n are believed to arrange along the GB within the octatomic rings.

**Figure 3:**
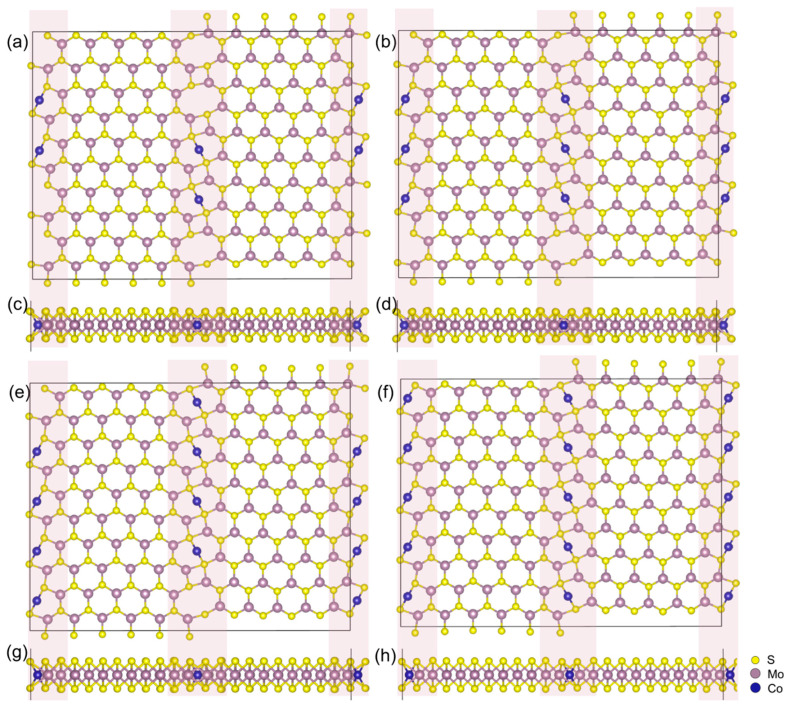
Top and side views for TM atomic chain assembled on monolayer MoS_2_ GB taking Co as an example: a direction, n = 2 for (**a**,**c**); a direction, n = 3 for (**b**,**d**); a direction, n = 4 for (**e**,**g**); a direction, n = 5 for (**f**,**h**). The atoms at the grain boundary are covered by pink rectangles.

The atomic structures of multiple TM atoms from monoatomic to pentatomic assembly at the MoS_2_ GB along direction a are illustrated in Figure 3, showing an example of Co, whose cell box constants are listed in Supporting Information Table S4. The pentatomic assembly suggests full occupation at the constructed grain boundary model and represents the formation of a TM atomic chain in the periodic GB structure. The concerned TM assembling at the GB region presents a similar pattern to that of Co, where slight lattice expansion is observed on the octatomic rings due to the TM interstitials. The GB structure maintains highly symmetric character with the increasement of the number of transition atoms (n = 2–5). Please note that n = 5 equals an infinite atomic chain due the periodic images.

**Figure 4:**
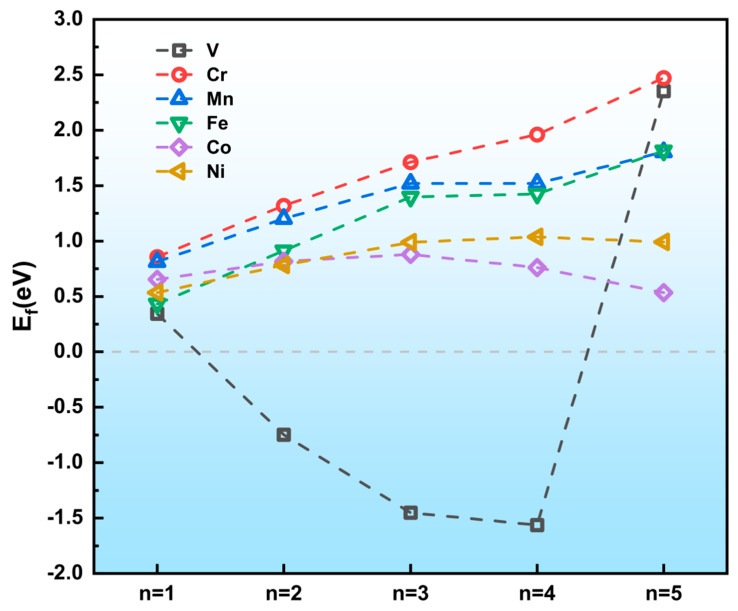
Formation energies of the building blocks of TM_n_/GB@MoS_2_ (n = 1–5). The concerned TM atoms are assembled on MoS_2_ GB along the a direction.

Thermodynamic energetic evaluations of the TM atomic chains can be found in Figure 4. It is obvious that the formation energies of the V_n_/GB@MoS_2_ (n = 2–4) systems gradually decrease, as the atomic chain extending along the a direction and the values are all negative, which indicates a spontaneous tendency of formation. Compared to the V-doped systems, the formation energies of other TM/GB@MoS_2_ systems remain as positive values when the TM atoms assemble into longer chains. The formation energies of Co and Ni atomic chains present a decreased tendency with an increasing number of atoms, while the formation energies of Cr, Fe, and Mn increase almost linearly to the atomic number of the chains.

### 3.3. Magnetic and Electronic Properties of Adsorbed Atomic Chains on GB

Magnetic moments can be induced into the GB system with the formation of TM atomic chains, and the corresponding magnetic moments are summarized in Table 2. The distribution of the local magnetic moments in some TM/GB@MoS_2_ systems can be observed in Supporting Information Tables S5 and S6 and Figure S1. The total magnetic moments of some V-doped systems are equal to zero due to the cancellation of the opposing orientation from structural relaxations. Despite that the formation energy of V_5_/GB@MoS_2_ shows a sudden increase, vanadium doping can result in a magnetic quaternary atomic chain at the MoS_2__4|8ud GB. During the formation of a chain of five V atoms, the size of the V atomic chain changes from a finite length into an infinite length. The interactions between V atoms lead to a severe lattice distortion of the eight-membered ring due to the larger atomic radius, resulting in a relatively unstable atomic structure, and thus, a sudden change in the formation energy of the V system occurs when the infinite chain forms.

**Figure 5:**
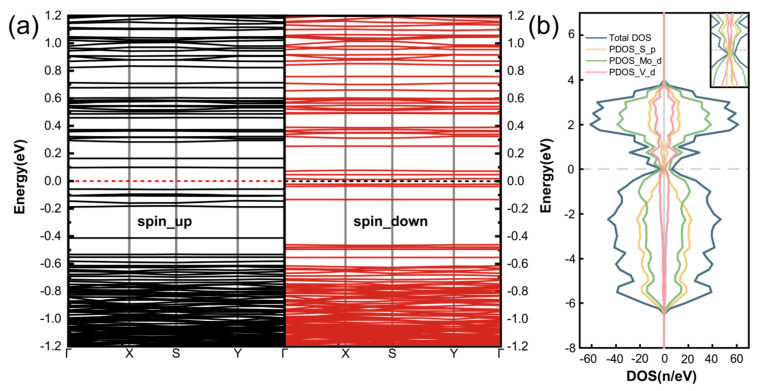
Band structure and density of states distribution of V_4_/GB@MoS_2_: (**a**) spin-up and spin-down band structure; (**b**) total density of states and orbital-projected density of states of grain boundary atoms, with the enlarged view of the density of states near the Fermi level shown in the upper right corner.

To understand the electronic and magnetic properties of the TM/GB@MoS_2_, the band structure and density of states of the V_4_/GB@MoS_2_ system are analyzed, as shown in Figure 5. The V_4_/GB@MoS_2_ system demonstrates an obvious spin split near the Fermi level, which results in a magnetic moment of 0.64 μ_B_ of the V_4_/GB@MoS_2_ system. The calculations show that the total bandgap of the system is 0.03 eV, which is typically negligible due to the thermal energy at room temperature. The V_4_/GB@MoS_2_ system is predicted to behave a semimetal with semiconducting properties at room temperature, which is conducive to enhancing the carrier mobility of the material. Combining the analysis of the TDOS and the PDOS, by observing the enlarged view around the Fermi level, it can be seen that the main contributions to the total density of states spin splitting come from the Mo_d and V_4 orbitals. A strong interaction between the V atom chain and the Mo atoms in the octagonal rings of the MoS_2__4|8ud grain boundary is observed. The magnetism originates from the d-orbitals of V atoms and its nearby Mo atoms and p-orbitals of S atoms through p-d hybridization.

**Figure 6:**
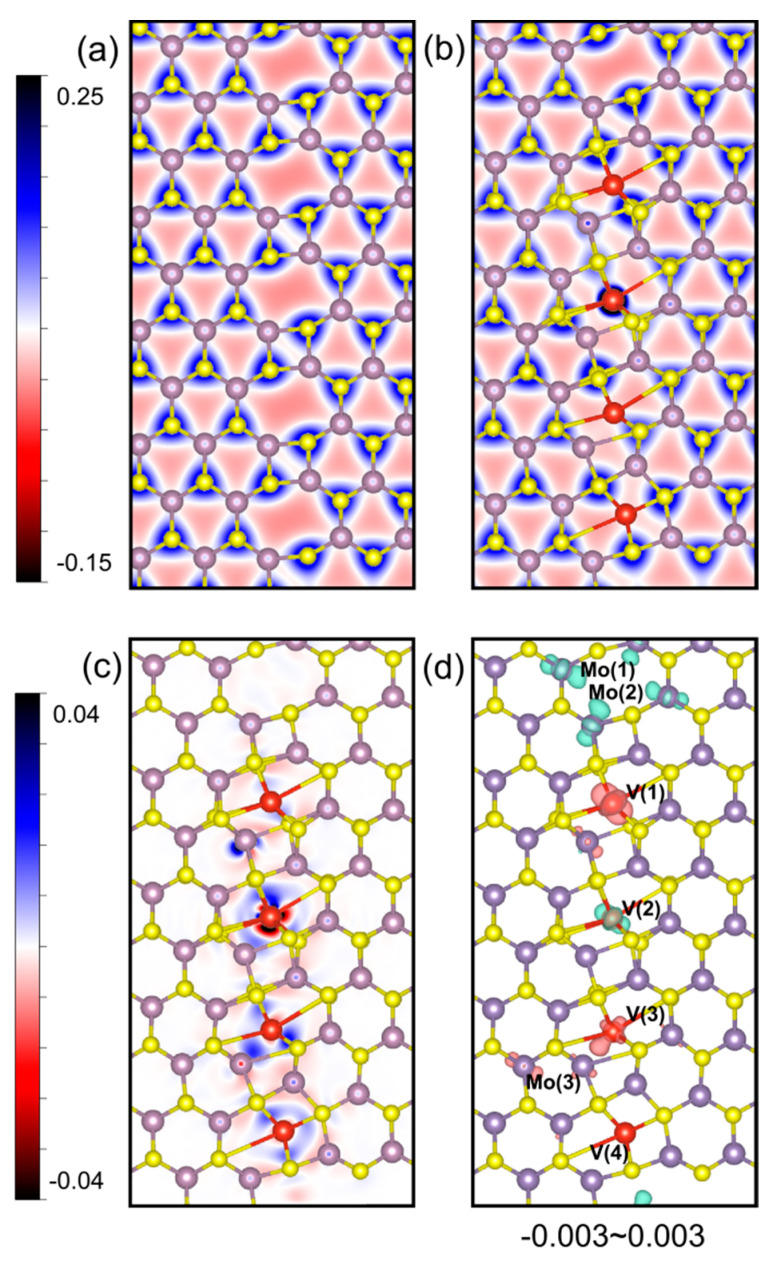
Charge density distribution maps of the MoS_2__4|8ud and V_4_/GB@MoS_2_ systems from the (001) cross-section: (**a**) MoS_2__4|8ud system; (**b**) V_4_/GB@MoS_2_ system. The characterization of charge density is determined by the numerical values on the axes and the corresponding colors in the figure. (**c**) Differential charge density distribution maps of V_4_/GB@MoS_2_ systems on the (001) surface. The red region represents electron loss, and the blue region represents electron gain. (**d**) Spin density distribution maps of V_4_/GB@MoS_2_ systems on the (001) surface. The red and blue iso-surfaces indicate the spin-up and -down densities with range values from −0.003 to 0.003.

To further understand the electronic and magnetic properties, electron distributions and spin density distributions of the MoS_2__4|8ud and V_4_/GB@MoS_2_ systems are plotted in Figure 6. The introduction of TM atoms in the overall structure of TM atomic chains will alter the charge density distribution at the grain boundaries. With the higher electronegativity of the doped TM atoms, electrons transfer from Mo and S atoms to the TM atoms. The charge redistribution between V(1–4) and the nearby S atoms may vary due to lattice distortion caused by TM chains. Spins are aligned around the transition atoms in the octatomic ring and its surrounding Mo and S atoms. Magnetism originates from the magnetic coupling of a V atom and its nearest-neighboring Mo and S atoms for a single TM interstitial in the GB. For multi-atomic TM chains, the distribution pattern of spin moments around each TM atom varies with the bond angles between the TM and S atoms, as well as the lattice symmetry of the GB model. Much more intense charge transfer happens between V and nearby S atoms, with a smaller bond angle of S-V-S. The local magnetic moments of V atoms labeled V(1) to V(4) in the V_4_/GB@MoS_2_ system are calculated to be 0.34 μ_B_, −0.12 μ_B_, 0.08 μ_B_, and −0.01 μ_B_, respectively. The magnetic moment is primarily contributed by the V(1–3) and Mo(1–3) atoms. The asymmetric distribution of the spins can be owed to the lattice distortion. After relaxation, the octagonal rings occupied by the V(1–4) atoms all experienced varying degrees of lattice distortion, with the central positions of the V atoms also shifting to different extents, caused by a boundary effect. Due to changes in the S-V-S bond angle, the magnetization intensity between the V atoms on the four-atom chain and the octagonal rings differs, which can be visually observed in Figure 6 and Table S6. Combined with Figure 5, in the self-assembled system of the V_4_ chain, the primary interactions are the spin–orbit coupling effects between the V_d orbitals and the Mo_d and S_p orbitals. This means that magnetic coupling occurs between the d-d and d-p orbitals, which is also the source of the system’s magnetism.

## 4. Conclusions

In summary, energetic and magnetic properties of atomic chains formed by six common transition metal elements (V, Cr, Mn, Fe, Co, and Ni) doped with monolayer MoS_2_ with 4|8ud grain boundaries were studied by first-principles calculations. For the undoped 4|8ud grain boundary, it is not magnetic itself, and the total magnetic moment is 0. After the doping transition metal assembles an atomic chain along the 4|8ud grain boundary, the magnetism of the system will change greatly. The calculation results show that the atomic chains of V, Cr, Mn, Fe, Co, and Ni all show different degrees of magnetism when they are doped into the 4|8ud-type grain boundary in the a direction. Among them, V atoms most easily fill the gaps along the octagonal rings in the MoS_2__4|8ud grain boundary for self-assembly, forming the V_n_/GB@MoS_2_ (n = 2–4) systems, in which the V_4_/GB@MoS_2_ exhibits the lowest formation energy. Furthermore, in contrast to the V_2_/GB@MoS_2_ and V_3_/GB@MoS_2_, only the V_4_/GB@MoS_2_ system demonstrates magnetic moment characteristics. The results of this study are beneficial to tune the magnetic properties of MoS_2_ trough transition metal doping, providing theoretical guidance for the experimental preparation of new low-dimensional microelectronic devices for information storage and transmission, which expands the boundaries of the development of spintronic devices based on low-dimensional materials.

## Figures and Tables

**Table 1:** Formation energy (eV) and total magnetic moment (μ_B_) of TM_2_/GB@MoS_2_ formed by filling the gaps along the a, b, and c directions.

TM_2_/GB@MoS_2_	Direction	E_f_ (eV)	Total Magnetic Moment (μ_B_)
Ni_2_/GB@MoS_2_	a	0.78	2.03
	b	1.73	1.99
	d	2.13	4.00
Mn_2_/GB@MoS_2_	a	1.20	8.92
	b	2.96	3.94
	c	4.60	6.00

**Table 2:** The calculated results of spin magnetic moment (μ_B_) of transition atom chains (V, Cr, Mn, Fe, Co, and Ni).

		V	Cr	Mn	Fe	Co	Ni
a direction	n = 1	2.19	4.00	3.41	0	2.00	0
	n = 2	0	8.000	8.92	4.00	3.45	2.03
	n = 3	0	12.00	13.61	8.00	0.80	0
	n = 4	0.64	12.02	19.71	8.000	0	−0.04
	n = 5	17.32	20.00	28.00	16.00	−0.48	0

## Data Availability

The data presented in this study are available on request from the corresponding author.

## Supplementary Materials

The following supporting information can be downloaded at: https://www.mdpi.com/article/10.3390/nano14242043/s1.
