# Supplementary material for: Formation and Magnetic Properties of Transition Metal Atomic Chains on Monolayer MoS2 Grain Boundaries: A First-Principles Study
Source: Nanomaterials (Basel). 2024 Dec 20;14(24):2043. doi: 10.3390/nano14242043 (PMC11678194; doi:10.3390/nano14242043)
Supplement: Supplementary file 1 [file nanomaterials-14-02043-s001.zip › nanomaterials-3340589-supplementary.pdf]

Supporting Information

Formation and Magnetic Properties of Transition Metal Atomic Chains on Monolayer MoS_2 Grain Boundaries: A First-Principles Study

Zhiyuan Li [1], Shuqing Yang [1] and Yiren Wang [1,2,*]

1 Key Laboratory for Nonferrous Materials (MOE), School of Materials Science and Engineering, Central South University, Changsha 410083, China;

8204201406@csu.edu.cn (Z.L.); 8204181110@csu.edu.cn (S.Y.)

2 National Key Laboratory for Powder Metallurgy, Central South University, Changsha 410083, China

* Correspondence: yiren.wang@csu.edu.cn

Table S1. The calculated results of substituted formation energies (E_f/eV), binding energies (E_b/eV) and total magnetic moment (μB) of the doped defects (V, Cr, Mn, Fe, Co and Ni) from " Dopants and grain boundary effects in monolayer MoS_2: a first-principles study "

		Ef		Eb	Total Magnetic Moment
		S-rich	S-poor		
V_{Mo}	perfect[a]	-2.37	0.23	-6.27	0
	sub_1	-2.66	-0.06	-5.10	0
	sub_2	-2.66	-0.06	-5.34	0
Cr_{Mo}	perfect[a]	-1.88	0.72	-5.78	0
	sub_1	-2.17	0.43	-4.60	0
	sub_2	-2.01	0.59	-4.45	0
Mn_{Mo}	perfect[a]	-0.75	1.85	-4.65	1.00
	sub_1	-1.92	0.68	-4.36	0.18
	sub_2	-1.58	1.02	-4.01	0.46
Fe_{Mo}	perfect[a]	0.40	3.00	-3.51	1.86
	sub_1	-1.9	1.21	-3.83	0
	sub_2	-0.67	1.93	-3.11	0
Co_{Mo}	perfect[a]	1.35	3.95	-2.55	2.64
	sub_1	-0.51	2.09	-2.95	1.10
	sub_2	0.15	2.75	-2.29	0
Ni_{Mo}	perfect[a]	1.74	4.34	-2.16	0
	sub_1	-0.12	2.48	-2.56	0
	sub_2	0.92	3.52	-1.52	0

Table S2. After relaxation, the adsorption energy of transition metal gas phase atoms μ_i, the total energy of each doping system E_{TM}, and the total energy of the pure MoS_2 4|8ud grain boundary $E_{perfect}$ are listed below.

Energy (eV)		V	Cr	Mn	Fe	Co	Ni
μ_i		-9.12	-9.62	-9.16	-8.30	-7.11	-5.57
E_{TM}	n=1	-2396.39	-2396.37	-2395.55	-2394.59	-2391.76	-2388.92
	n=2	-2416.81	-2414.69	-2413.09	-2410.23	-2405.64	-2399.56
	n=3	-2436.45	-2433.14	-2430.78	-2425.87	-2419.73	-2410.29
	n=4	-2454.90	-2451.88	-2449.10	-2442.42	-2434.19	-2421.33
	n=5	-2465.30	-2470.10	-2466.86	-2458.24	-2448.86	-2432.56
$E_{perfect}$		-2378.85					

Table S3. After relaxation, formation energies of each doping system are listed below.

E_f (eV)	V	Cr	Mn	Fe	Co	Ni
n=1	0.34	0.86	0.81	0.43	0.65	0.53
n=2	-0.75	1.32	1.20	0.91	0.82	0.78
n=3	-1.45	1.71	1.52	1.40	0.88	0.99
n=4	-1.56	1.96	1.52	1.43	0.76	1.04
n=5	2.35	2.47	1.80	1.82	0.53	0.99

Table S4. The system parameters of Co_n/GB@MoS_2, n=2,3,4 and 5.

Co_n/GB@MoS_2	a (Å)	b (Å)	c (Å)	α(°)	β(°)	γ(°)
n=2	27.7	35.8	20.0	90.0	90.0	90.1
n=3	27.7	35.9	20.0	89.9	90.0	90.1
n=4	27.7	35.9	19.9	90.0	89.9	90.0
n=5	27.8	36.0	19.8	89.9	90.0	90.0

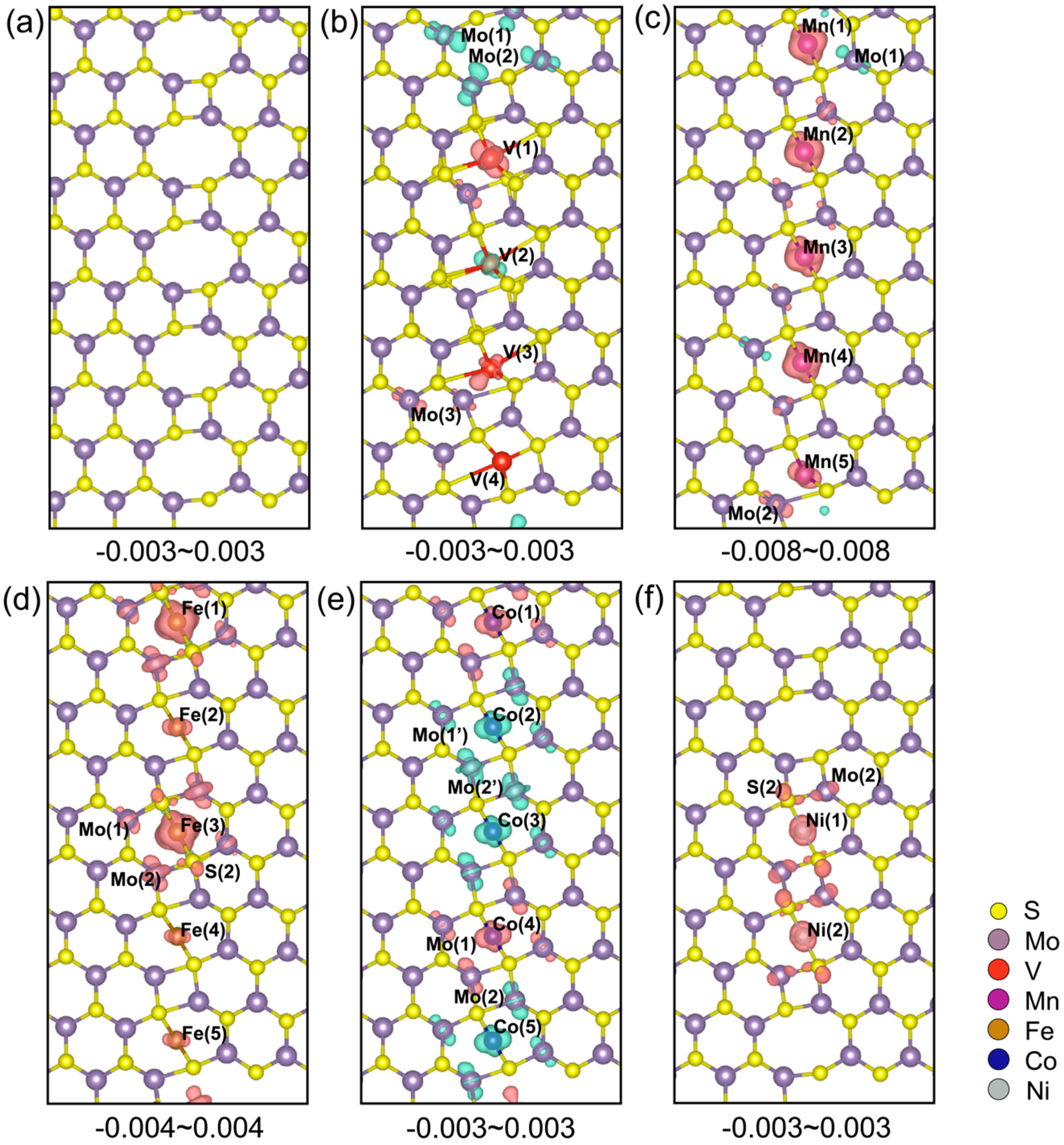

Figure S1. Spin density distribution maps of the MoS2_4|8 grain boundary and TM/GB@MoS_2 systems: (a) MoS_2_4|8 ud grain boundary; (b) V_4/GB@MoS_2; (c) Mn_5/GB@MoS_2; (d) Fe_5/GB@MoS_2; (e) Co_5/GB@MoS_2; (f) Ni_2/GB@MoS_2; the contour range values are shown below the corresponding spin density maps.

Table S5. Distribution of local magnetic moments in the TM/GB@MoS_2 systems, which can be found in Figure S1, are listed below. The atomic numbers can be found in Figure S1.

System	Atom	Local Magnetic Moment(μ_B)	Atom	Local Magnetic Moment(μ_B)
V_4/GB@MoS_2	V(1)	0.34	Mo(1)	-0.11
	V(2)	-0.12	Mo(2)	-0.11
	V(3)	0.08	Mo(3)	0.06
	V(4)	-0.01		
Mn_5/GB@MoS_2	Mn(1)	2.75	Mo(1)	-0.13
	Mn(2)	2.56	Mo(2)	0.12
	Mn(3)	2.48		
	Mn(4)	2.83		
	Mn(5)	0.56		
Fe_4/GB@MoS_2	Fe(1)	2.83	Mo(1)	0.1
	Fe(2)	0.1	Mo(2)	0.16
	Fe(3)	2.82	S(2)	0.04
	Fe(4)	0.05		
	Fe(5)	0.06		
Co_4/GB@MoS_2	Co(1)	0.27	Mo(1)	-0.05
	Co(2)	-0.31	Mo(2)	-0.11
	Co(3)	-0.31	Mo(1’)	0.04
	Co(4)	0.27	Mo(2’)	0.06
	Co(5)	-0.31		
Ni_2/GB@MoS_2	Ni(1)	0.55	Mo(2)	0.07
	Ni(2)	0.55	S(2)	0.03

Table S6. Distribution of local magnetic moments (μB) of the transition metals in some TM/GB@MoS_2 systems are listed below. The number of the TM atoms is listed in Figure S1.

Systems		TM atoms				
		1	2	3	4	5
V	n=4	0.34	-0.12	0.08	-0.01	0.01
	n=1	2.20	1.99			
	n=2	2.21	2.10	1.96	1.96	
Cr	n=3	2.09	1.99	2.13	1.97	1.96
	n=4	2.09	1.94	1.95	2.12	2.05
	n=5	1.92	1.90	1.91	1.90	1.90
	n=1	1.92	2.67			
	n=2	2.26	2.26	2.47	2.44	
Mn	n=3	2.18	2.54	2.13	0.28	2.62
	n=4	0.25	2.63	2.61	2.18	2.27
	n=5	0.56	2.83	2.48	2.56	2.75
	n=1	0.00	0.00			
	n=2	0.00	0.00	0.06	2.83	
Fe	n=3	0.00	0.00	0.00	2.84	0.15
	n=4	0.00	0.05	2.82	0.04	0.00
	n=5	0.06	0.05	2.82	0.10	2.83
	n=1	-0.03	-0.35			
	n=2	0.30	0.22	0.40	0.42	
Co	n=3	-0.18	0.09	0.07	-0.26	-0.11
	n=4	0.18	0.00	0.04	0.16	0.17
	n=5	-0.31	0.27	-0.31	-0.31	0.27
	n=1	0.79	0.79			
	n=2	0.55	0.55	0.55	0.55	
Ni	n=3	0.69	0.47	0.67	0.71	-0.64
	n=4	0.70	0.64	-0.62	0.70	-0.70
	n=5	0.49	0.60	-0.63	-0.63	0.60

Table S7. The bond angle (°) between the V atoms and S atoms in Figure S1 is listed by atomic number in Figure S1.

	S-V-S(1)	S-V-S(2)	S-V-S(3)	S-V-S(4)
V(1)	72.59	60.09	86.69	52.60
V(2)	73.47	59.78	90.18	51.61
V(3)	75.23	59.09	94.51	51.87
V(4)	76.42	49.86	78.86	70.37

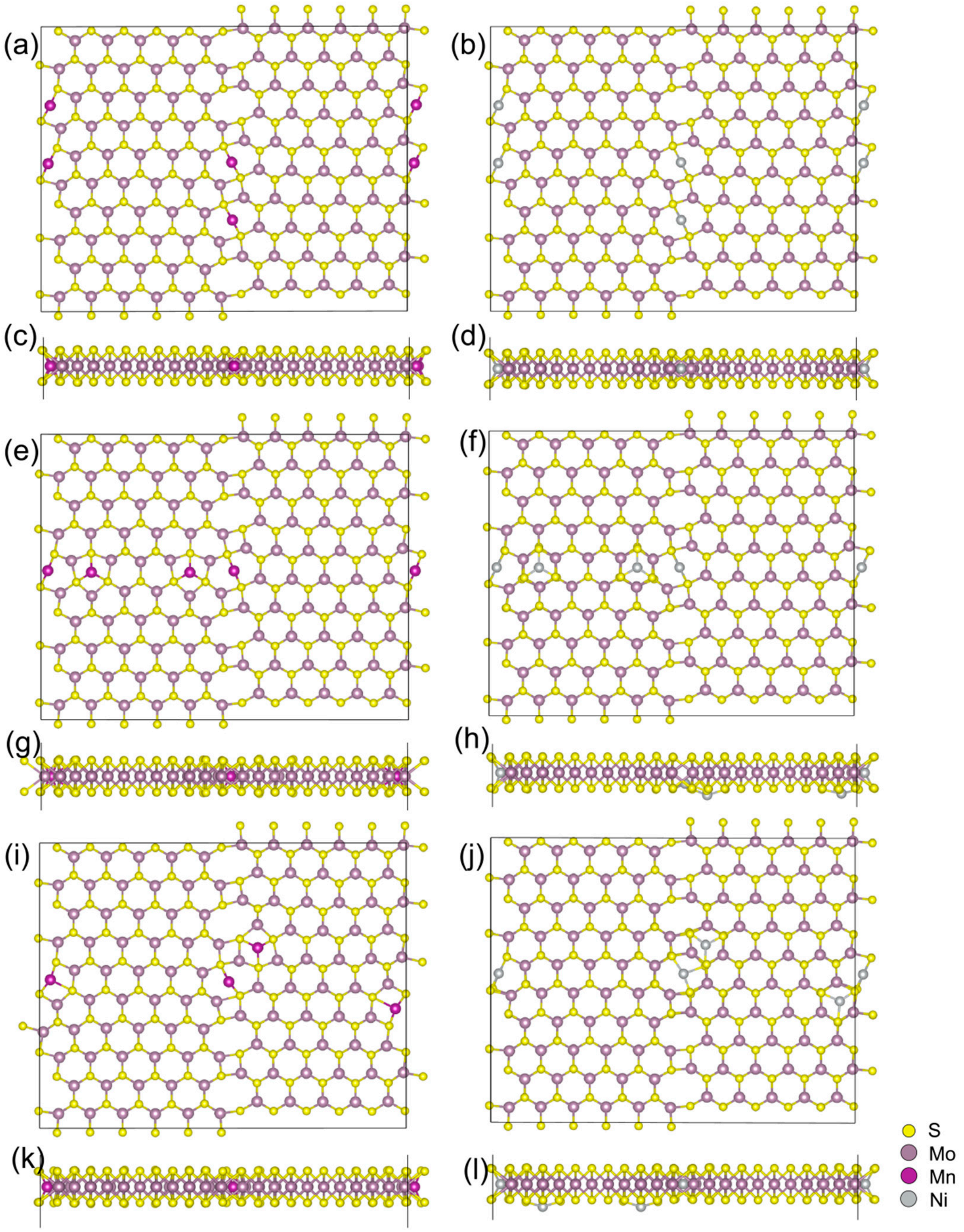

Figure S2. Top view and front view of the relaxed structure of TM_2/GB@MoS_2 self-assembled along directions a, b, and c. (a)(c) are for Mn_2/GB@MoS_2 along the direction a; (e)(g) are for Mn_2/GB@MoS_2 along the direction c; (i)(k) are for Mn_2/GB@MoS_2 along the direction b; (b)(d) are for Ni_2/GB@MoS_2 along the direction a; (f)(h) are for Ni_2/GB@MoS_2 along the direction c; (j)(l) are for Ni_2/GB@MoS_2 along the direction b.
